# Cost-effectiveness and health impact of lung cancer screening with low-dose computed tomography for never smokers in Japan and the United States: a modelling study

**DOI:** 10.1186/s12890-021-01805-y

**Published:** 2022-01-08

**Authors:** Akiko Kowada

**Affiliations:** 1 Department of Occupational Health, Kitasato University Graduate School of Medical Sciences, 1-15-1 Kitasato, Minami-ku, Sagamihara, Kanagawa 252-0373 Japan; 2 Health Sciences University of Hokkaido, 1757 Kanazawa, Tobetsu-cho, Ishikari-gun, Hokkaido 061-0293 Japan

**Keywords:** Low-dose computed tomography, Never smoker, Adenocarcinoma, Lung cancer, Health economics

## Abstract

**Background:**

Never smokers in Asia have a higher incidence of lung cancer than in Europe and North America. We aimed to assess the cost-effectiveness of lung cancer screening with low-dose computed tomography (LDCT) for never smokers in Japan and the United States.

**Methods:**

We developed a state-transition model for three strategies: LDCT, chest X-ray (CXR), and no screening, using a healthcare payer perspective over a lifetime horizon. Sensitivity analyses were also performed. Main outcomes were costs, quality-adjusted life-years (QALYs), life expectancy life-years (LYs), incremental cost-effectiveness ratios (ICERs), and deaths from lung cancer. The willingness-to-pay level was US$100,000 per QALY gained.

**Results:**

LDCT yielded the greatest benefits with the lowest cost in Japan, but the ICERs of LDCT compared with CXR were US$3,001,304 per QALY gained for American men and US$2,097,969 per QALY gained for American women. Cost-effectiveness was sensitive to the incidence of lung cancer. Probabilistic sensitivity analyses demonstrated that LDCT was cost-effective 99.3–99.7% for Japanese, no screening was cost-effective 77.7% for American men, and CXR was cost-effective 93.2% for American women. Compared with CXR, LDCT has the cumulative lifetime potential for 60-year-old Japanese to save US$117 billion, increase 2,339,349 QALYs and 3,020,102 LYs, and reduce 224,749 deaths, and the potential for 60-year-old Americans to cost US$120 billion, increase 48,651 QALYs and 67,988 LYs, and reduce 2,309 deaths.

**Conclusions:**

This modelling study suggests that LDCT screening for never smokers has the greatest benefits and cost savings in Japan, but is not cost-effective in the United States. Assessing the risk of lung cancer in never smokers is important for introducing population-based LDCT screening.

## Background

Lung cancer is the most commonly occurring cancer in men and the third most commonly occurring cancer in women worldwide. There were 2 million new lung cancer cases in 2018 in the world [1]. Several trials strongly suggested that low-dose computed tomography (LDCT) screening efficiently detects early-stage lung cancer and reduces lung cancer mortality in heavy smokers. The National Lung Screening Trial (NLST) demonstrated a relative reduction in lung-cancer mortality of 20% in former and current heavy smokers ages 55 to 74 who underwent screening with LDCT, as compared with chest X-ray (CXR) [2, 3]. The Nederlands–Leuvens Longkanker Screenings Onderzoek (NELSON) trial showed that lung-cancer mortality among male former and current smokers with LDCT screening was significantly lower than those with no screening [4]. The US Preventive Services Task Force (USPSTF) recommends annual LDCT screening as a secondary prevention strategy for people aged 55 to 80 years with a 30-pack-year smoking history or those who are currently smoking or quit within 15 years [5]. Currently, LDCT screening in combination with tobacco cessation is recommended to improve the prognosis of lung cancer in current heavy smokers [6]. Several cost-effectiveness studies in heavy smokers also showed the superiority of LDCT screening [7–12].

Recently, as tobacco consumption has declined, the lung cancer mortality rate in men has continued to decline [13–15]. However, the proportion of never-smoking patients with non-small cell lung cancer (NSCLC) has been significantly increasing over 30 years from 15.9% in the 1970s to 32.8% in the 2000s [16–18]. Especially, the high proportion of lung cancer in female never smokers in Asia is predominant 61% in Eastern and 83% in Southern Asia, compared to 15% in the United States [19, 20].

In Japan, lung cancer is the third most common cancer in men, the fourth most common cancer in women, and the most common cause of cancer death that is 20% of all cancer deaths. In 2017, 22,471 lung cancer cases were diagnosed, and 74,328 lung cancer deaths were reported in 2018 [21]. According to Japanese guidelines for lung cancer screening, annual population-based lung cancer screening using CXR is recommended for all people over the age of 40 [22]. However, the early detection rate of lung cancer is low, and the delay in diagnosing and treating lung cancer leads to poor prognosis. The five-year survival rate of lung cancer is 29.5% for men and 46.8% for women in Japan [21]. Nearly 90% of lung cancer in never smokers are adenocarcinomas in Japan, which can be treated with curable surgery if detected early, making them suitable for detection with LDCT screening [23–25]. In the United States, about 60% of lung cancer in never smokers is also adenocarcinoma [26]. More effective lung cancer screening for never smokers is urgently needed to save lives from lung cancer. Population-based LDCT screening for never smokers has great potential to help detect significant numbers of very early-stage lung cancers, especially in Asia [23, 27]. Lung cancer screening using LDCT in never smokers is a major concern for the secondary prevention method of lung cancer. Cost-effectiveness regarding LDCT screening for never smokers warrants evaluation as a lung cancer control measure.

In this study, we assessed the cost-effectiveness of LDCT compared with CXR and no screening to implement the optimal lung cancer screening method for never smokers in Japan, one of the high-incidence countries, and the United States, one of the low-incidence countries.

## Methods

### Model design and structure

**Fig. 1:**
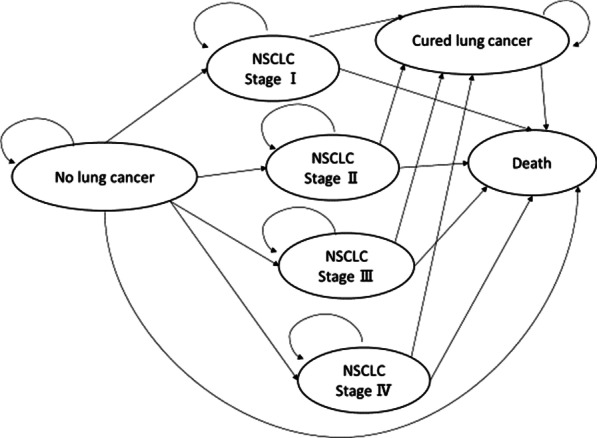
Schematic depiction of a Markov cycle tree in a state-transition model. Health states in the model are indicated with ovals. Over the course of a year-long model cycle, transitions between one health state and another may occur, which are indicated by pointing arrowheads. *NSCLC* non-small cell lung cancer

We developed a state-transition model for three strategies: LDCT, CXR, and no screening (Fig. 1). We targeted four hypothetical cohorts of 60-year-old male never smokers and 60-year-old female never smokers in Japan and the United States using a healthcare payer perspective over a lifetime horizon. The main outcomes were costs, quality-adjusted life-years (QALYs), life expectancy life-years (LYs), incremental cost-effectiveness ratios (ICERs), and deaths from lung cancer. A cycle length of one year was chosen. The half-cycle correction was applied. Costs and QALYs were discounted by 3% year [28]. We performed decision-analytical calculations using TreeAge Pro (TreeAge Software Inc., Williamstown, Massachusetts).

In the model for lung cancer screening using LDCT or CXR, the individual receives LDCT or CXR. If LDCT or CXR is true positive and lung cancer is diagnosed by subsequent chest CT bronchoscopy with biopsy of lung tissue, the individual receives the standard treatment of lung cancer followed by the lung cancer treatment guidelines: surgery, or surgery combined with radiotherapy, or chemotherapy combined with radiotherapy, or chemotherapy combined with palliative care depending on the stage of cancer in Japan [29], and receives the standard treatment of lung cancer based on the Surveillance, Epidemiology, and End Results (SEER)‐Medicare database in the United States [30]. The individual with false positive results continues to undergo subsequent chest CT bronchoscopy with a biopsy of lung tissue but has negative results and no treatment of lung cancer. If LDCT or CXR is true negative, the individual returns to follow-up screening. The false negative results in lung cancer patients lead to LDCT or CXR in the following year. In any of the states, all individuals were at risk of all-cause mortality. We assume that the small cell lung cancer rate in never smokers is 0% in the model [31].

In no screening, we assume the stage detection proportions of lung cancer in Japanese from Japanese cancer statistics and those in Americans from literature [30, 32].

As this was a modelling study with all inputs and parameters derived from the published literature, cancer statistics, and vital statistics, ethics approval was not required.

### Costs, clinical probabilities, epidemiological parameters

Costs for Japanese never smokers were based on the direct costs of screening tests and cancer treatments from the Japanese national fee schedule [33], and were adjusted to 2019 Japanese yen, using the medical care component of the Japanese consumer price index and were converted to US dollars, using the Organisation for Economic Co-operation and Development (OECD) purchasing power parity rate in 2020 (US$1 = ¥103.4) [34]. The indirect costs, such as visits to inpatients and outpatients, the infrastructure, and medical staffs, were excluded. Costs for American never smokers were based on Medicare [35–37]. All costs were discounted by 3% [28, 38].

Clinical probabilities and epidemiological parameters were collected using MEDLINE from 2001 to August 7, 2021, Japanese cancer statistics, and SEER cancer statistics to estimate input parameters for the models (Table 1). The incidence of lung cancer, lung cancer mortality specific to lung cancer stage, stage detection proportion of lung cancer-specific to the screening methods, and the other mortality rate were estimated by the literature, cancer statistics, vital statistics, and life tables [16, 18, 21, 23, 26, 30, 32, 39, 40]. Since we hypothesized that the diagnostic capability of radiologists in both countries would be equal, we assumed that the stage detection proportion of lung cancer in LDCT and CXR screening was the same in both countries. The sensitivity and specificity of LDCT and CXR screenings were derived from the literature [41]. The adherence rate of the available screening methods was assumed to be 100%. We considered the cumulative increased radiogenic risk of cancer from repeated annual LDCT in the model [42, 43].

**Table 1:** Baseline estimates for selected variables in never smokers

Variable	Baseline value	Sensitivity analysis range	Reference
Incidence of lung cancer in 60-year-old never smokers in Japan			
Women	0.0152	0.001–0.03	[23]
Men	0.009	0.001–0.03	
Stage-specific 5-year survival rate in Japan			
Stage I	0.812	0.7–0.85	[21]
Stage II	0.463	0.3–0.6	
Stage III	0.223	0.15–0.5	
Stage IV	0.051	0.01–0.1	
Stage detection proportions of lung cancer in no screening in Japan			
Stage I	0.22	0.1–0.6	[32]
Stage II	0.06	0–0.2	
Stage III	0.21	0.1–0.3	
Stage IV	0.51	0.3–0.7	
Costs in Japan, US$			
CXR	28.5	14.3–57.0	[33]
LDCT	195.7	97.9–391.4	
Bronchoscopy with CT-guided lung biopsy	906.2	453.1–1812.4	
Treatment of lung cancer, Stage I	25,835	12,918–51,670	
Treatment of lung cancer, Stage II	37,758	18,879–75,516	
Treatment of lung cancer, Stage III	48,688	24,344–97,376	
Treatment of lung cancer, Stage IV	264,308	132,154–528,616	
Incidence of lung cancer in 60-year-old never smokers in the United States			
Women	0.000207	0.000135–0.000311	[26]
Men	0.000137	0.00009–0.000215	
Stage-specific 5-year survival rate in the United States			
Stage I	0.75	0.7–0.85	[16, 40]
Stage II	0.53	0.3–0.6	
Stage III	0.41	0.15–0.5	
Stage IV	0.07	0.01–0.1	
Stage detection proportions of lung cancer in no screening in the United States			
Stage I	0.24	0.1–0.6	[30]
Stage II	0.07	0–0.2	
Stage III	0.28	0.1–0.3	
Stage IV	0.41	0.3–0.7	
Costs in the United States, US$			
CXR	42.3	21.2–84.6	[35–37]
LDCT	254.6	127.3–509.2	
Bronchoscopy with CT-guided lung biopsy	681.0	340.5–1362.0	
Treatment of lung cancer, Stage I	20,984	10,492–41,968	
Treatment of lung cancer, Stage II	20,984	10,492–41,968	
Treatment of lung cancer, Stage III	37,987	18,994–75,974	
Treatment of lung cancer, Stage IV	82,601	41,301–165,202	
Stage detection proportions of lung cancer in CXR screening in Japan and the United States			
Stage I	0.61	0.4–0.7	[39]
Stage II	0.07	0–0.2	
Stage III	0.17	0.1–0.3	
Stage IV	0.15	0.1–0.3	
Stage detection proportions of lung cancer in LDCT screening in Japan and the United States			
Stage I	0.96	0.8–1.0	[23]
Stage II	0.01	0–0.1	
Stage III	0.03	0–0.1	
Stage IV	0.01	0–0.1	
Accuracies (%)			
Sensitivity of CXR	73.5	67.2–79.8	[41]
Specificity of CXR	91.3	91.0–91.6	
Sensitivity of LDCT	93.8	90.6–96.3	
Specificity of LDCT	73.4	72.8–73.9	
Utilities			
Healthy	1	N/A	[44]
Stage I lung cancer	0.87	0.7–0.9	
Stage II lung cancer	0.87	0.7–0.9	
Stage III lung cancer	0.77	0.6–0.8	
Stage IV lung cancer	0.57	0.3–0.6	
Cured lung cancer	0.9	0.7–0.9	
Dead	0	N/A	
Cumulative increased radiogenic risk of cancer from repeated annual LDCT (%)			
Women	0.30	N/A	[42]
Men	0.13	N/A	

*LDCT* low-dose computed tomography, *CXR* chest X-ray, *N/A* not applicable

### Health state utilities

The following seven health states were included to represent possible clinical states in the Markov model: No lung cancer, stage I in non-small cell lung cancer (NSCLC), stage II in NSCLC, stage III in NSCLC, stage IV in NSCLC, cured lung cancer, and death (Fig. 1). Health state utilities were sourced from the literature [44, 45]. The QALYs were calculated by applying health state utility weights. The annual discounting of the utility was set at a rate of 3% [28, 38].

### Sensitivity analyses

We performed one-way sensitivity analyses to determine which strategy was more cost-effective when we tested a single variable over a wide range of possible values while holding all other variables constant. We used US$100,000 per QALY gained thresholds for the willingness-to-pay thresholds [46]. Probabilistic sensitivity analyses using a second-order Monte-Carlo simulation for 10,000 trials were conducted to assess the impact of uncertainty. The uncertainty had a beta distribution in clinical probabilities and accuracies, and a log-normal distribution in costs.

## Results

### Base-case analysis

In a base-case analysis, LDCT yielded the greatest benefits with the lowest costs in Japan (men, US$20,446, 17.8812 QALYs, 18.0655 LYs; women, US$30,065, 17.665 QALYs, 17.9603 LYs) (Table 2). In the United States, LDCT was not cost-effective compared to CXR and no screening, with the ICERs of LDCT over US$100,000 per QALY gained (Table 2).

**Table 2:** Results of the base-case analysis

Country	Gender	Strategy	Cost (US$)	Incremental cost (US$)	Quality-adjusted life-years (QALYs)	Incremental QALYs	ICER (US$/QALY gained)	Life expectancy life-years (LYs)	Incremental LYs	ICER (US$/LY gained)
Japan	Male	LDCT	20,446	–	17.8812	–	–	18.0655	–	–
		CXR	26,016	5,570	17.7508	− 0.1304	dominated	17.8955	− 0.1700	dominated
		No screening	73,315	52,869	17.2775	− 0.6036	dominated	17.4929	− 0.5726	dominated
	Female	LDCT	30,065	–	17.6650	–	–	17.9603	–	–
		CXR	41,420	11,354	17.4506	− 0.2144	dominated	17.6846	− 0.2756	dominated
		No screening	115,771	85,706	16.6997	− 0.9653	dominated	17.0419	− 0.9184	dominated
United States	Male	No screening	362	–	15.2610	–	–	15.2639	–	–
		CXR	929	568	15.2657	0.0047	121,806	15.2675	0.0036	155,850
		LDCT	4,497	3,567	15.2668	0.0012	3,001,304	15.2691	0.0016	2,205,027
	Female	No screening	606	–	16.9297	–	–	16.9346	–	–
		CXR	1,124	518	16.9384	0.0087	59,649	16.9416	0.0070	74,232
		LDCT	5,041	3,917	16.9403	0.0019	2,097,969	16.9442	0.0026	1,478,214

*LDCT* low-dose computed tomography, *CXR* chest X-ray, *ICER* incremental cost-effectiveness ratio, *dominated* less effective and more costly than others

### Sensitivity analyses

**Fig. 2:**
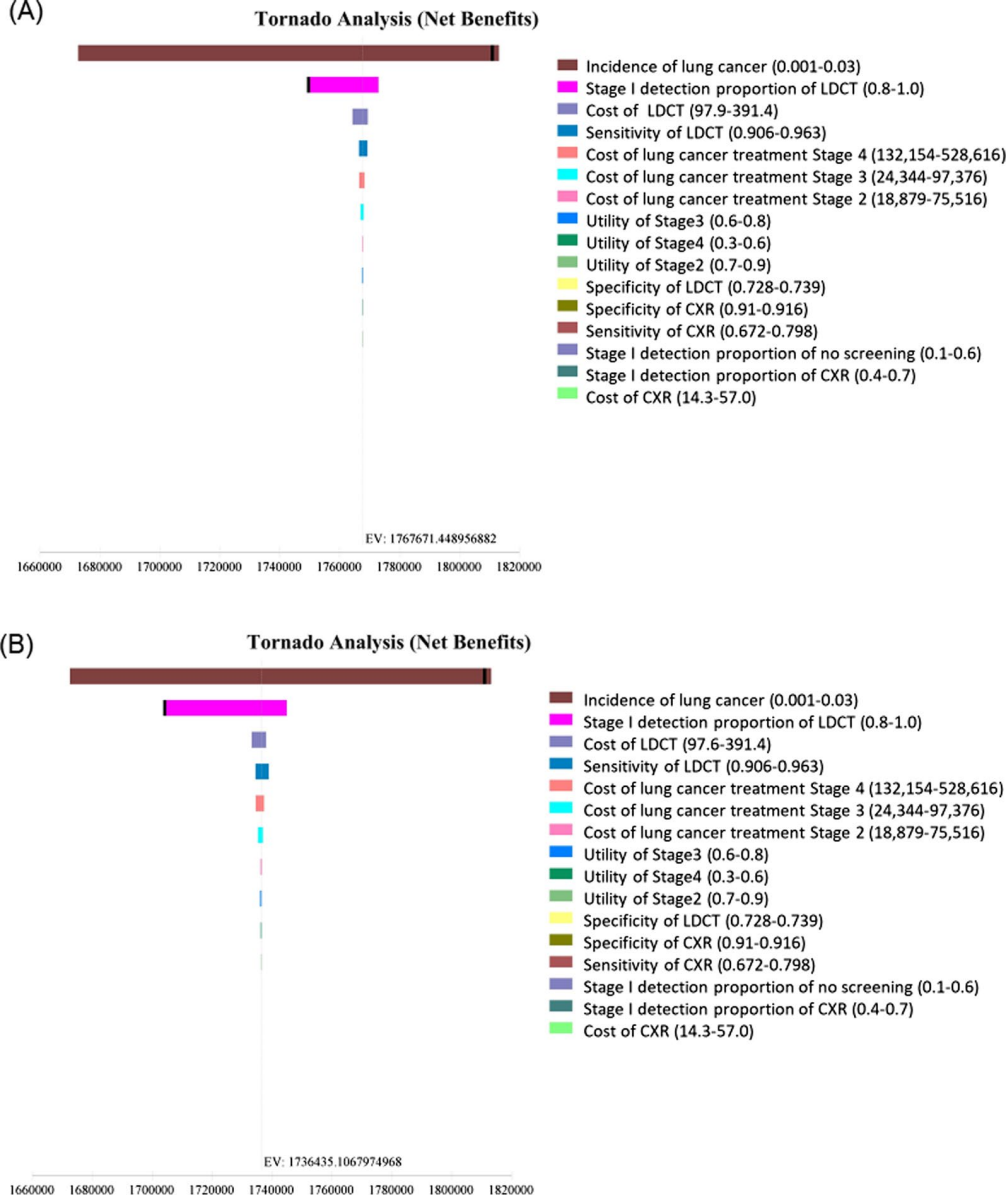

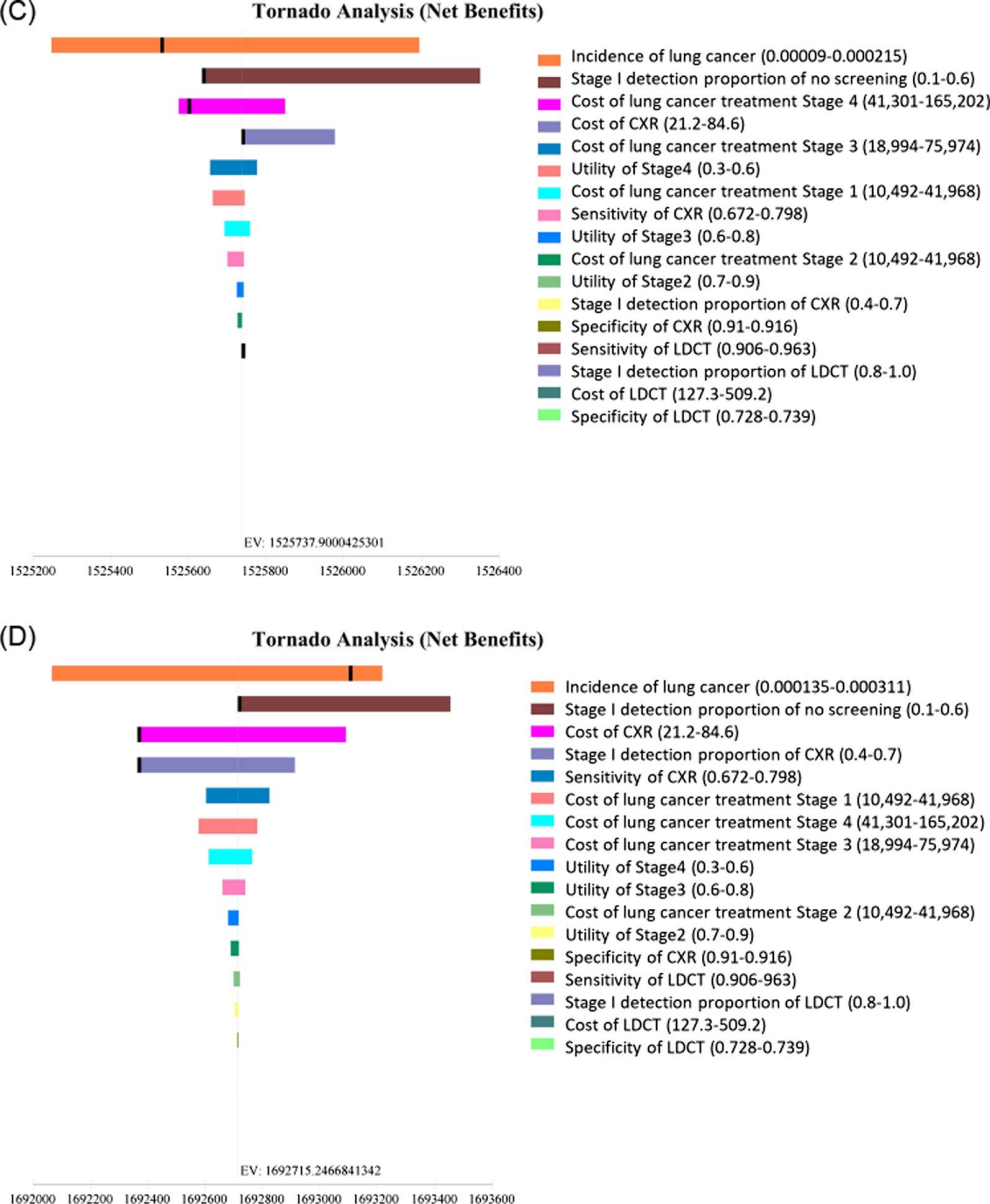
Tornado diagrams of one-way sensitivity analysis. **a** Male never smokers in Japan, **b** Female never smokers in Japan, **c** Male never smokers in the United States, **d** Female never smokers in the United States. Tornado bars display for each variable to show how the net benefit of the optimal alternate variables change. Heavy vertical lines identify threshold points. *LDCT* low-dose computed tomography, *CXR* chest X-ray, *EV* expected value

**Fig. 3:**
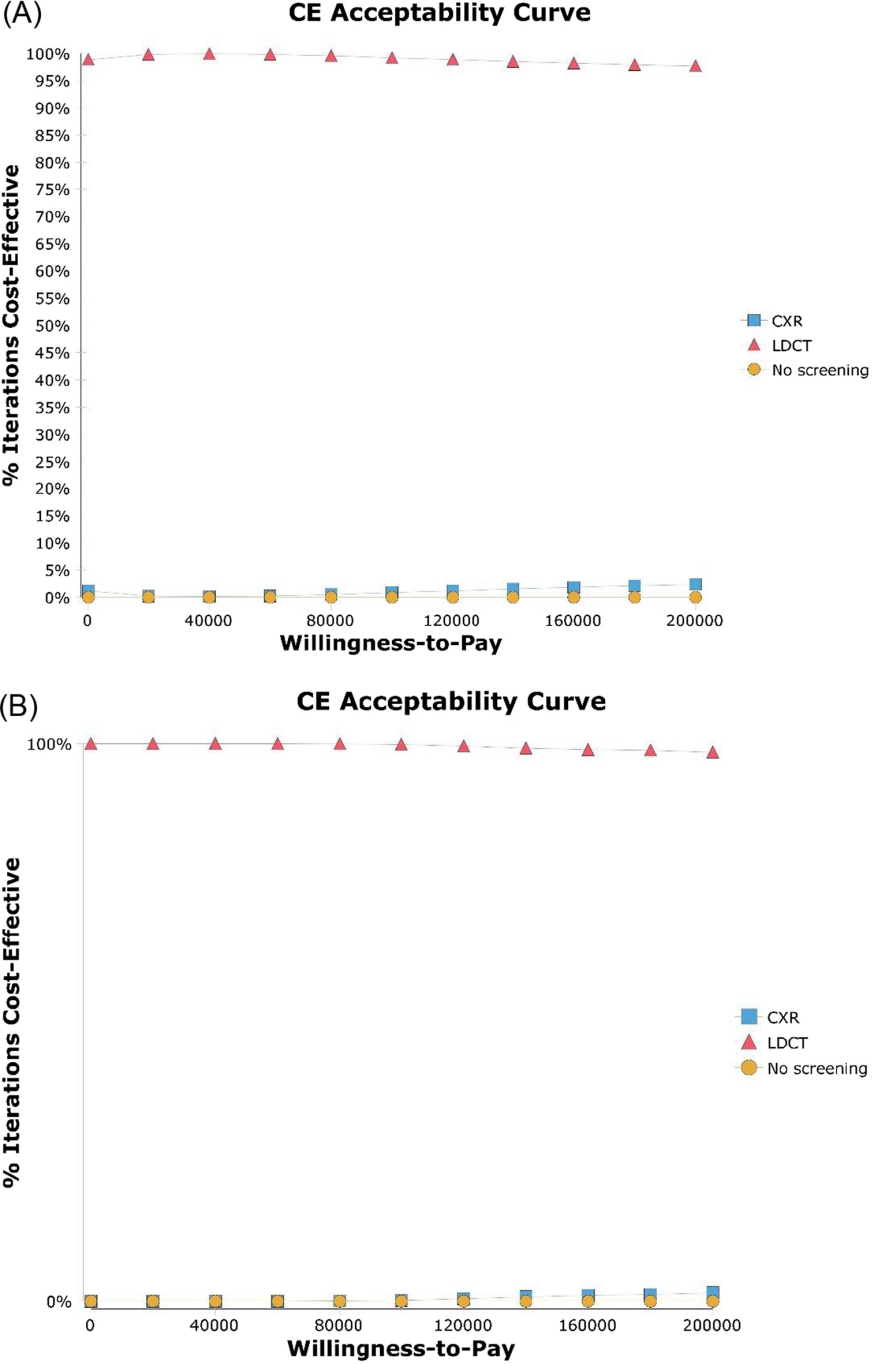

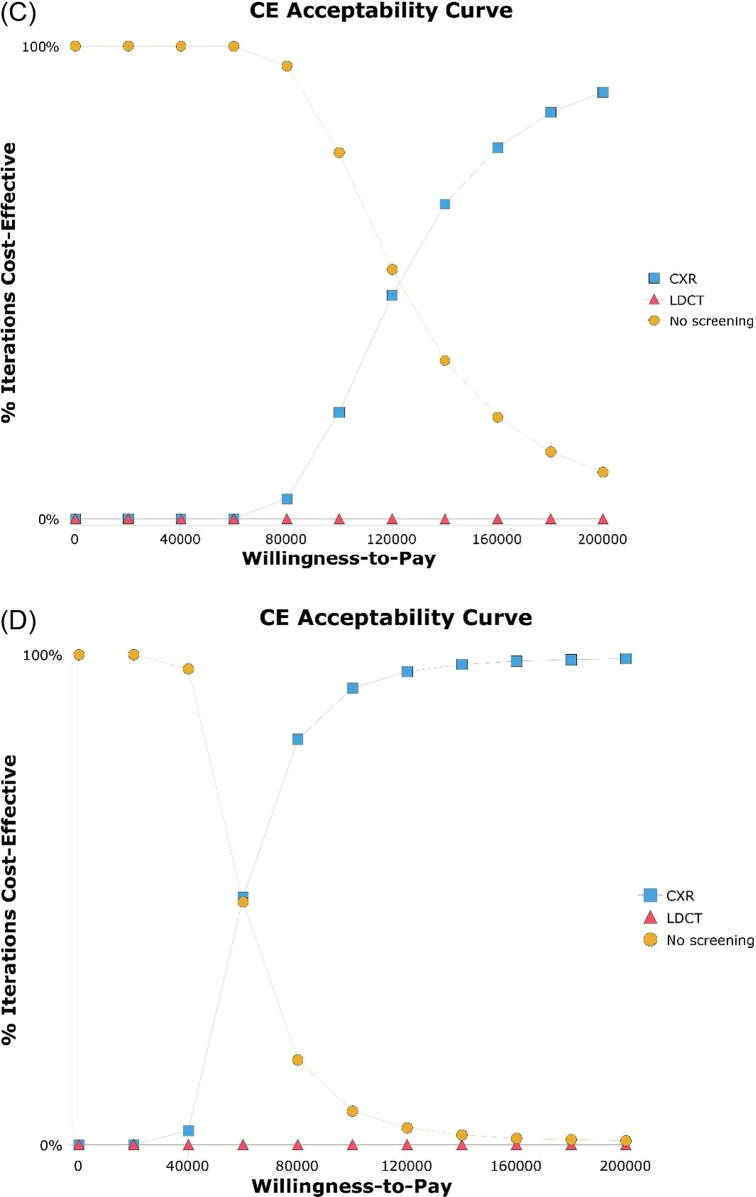
Cost-effectiveness acceptability curves. **a** Male never smokers in Japan, **b** Female never smokers in Japan, **c** Male never smokers in the United States, **d** Female never smokers in the United States. The probabilistic sensitivity analysis analyzed 10,000 simulations of models with randomly changed input parameters to understand how parameter uncertainty affects model results. The x-axis represents the willingness-to-pay threshold (US$ per QALY gained). *LDCT* low-dose computed tomography, *CXR* chest X-ray, *QALY* quality-adjusted life-year

Cost-effectiveness was sensitive to the incidence of lung cancer. In Japan, LDCT was more cost-effective than CXR when the incidence of lung cancer was higher than 0.00135 for men and 0.00134 for women (Fig. 2a, b). In the United States, CXR was more cost-effective than no screening when the incidence of lung cancer was over 0.00016 for men and 0.00014 for women (Fig. 2c, d). Probabilistic sensitivity analysis with 10,000 Monte Carlo simulations demonstrated that LDCT was cost-effective 99.3% for Japanese men (Fig. 3a) and 99.7% for Japanese women (Fig. 3b). The probability of no screening was 77.7% for American men (Fig. 3c) and the probability of CXR was 93.2% for American women at a willingness-to-pay level of US$100,000 per QALY gained (Fig. 3d).

### Economic and health outcomes

In the Markov cohort analysis, for 13.05 million 60-year-old Japanese never smokers, LDCT has a cumulative lifetime potential to save US$117 billion, increase 2,339,349 QALYs and 3,020,102 LYs, and reduce 224,749 deaths, compared with CXR. For 31.88 million 60-year-old American never smokers, LDCT has a cumulative lifetime potential to cost US$120 billion, increase 48,651 QALYs and 67,988 LYs, and reduce 2,309 deaths, compared with CXR (Table 3).

**Table 3:** Cumulative lifetime economic and health outcomes of LDCT

Country	Gender	60-year-old individuals (n)	Cost-saving (US$)	QALYs gain (QALYs)	LYs gain (LYs)	Deaths averted lung cancer (%)	Deaths averted lung cancer (n)
Compared with CXR							
Japan	Male	5,463,770	30,435,384,408	712,476	928,841	1.24	67,719
	Female	7,588,028	86,157,505,123	1,626,873	2,091,261	2.07	157,030
United States	Male	14,904,704	− 53,169,550,579	16,395	23,848	0.005	750
	Female	16,977,059	− 66,502,535,515	32,256	44,140	0.009	1,559
Compared with no screening							
Japan	Male	5,463,770	288,864,056,130	3,298,478	3,128,555	7.04	384,736
	Female	7,588,028	650,338,010,162	7,324,723	6,968,845	10.93	829,238
United States	Male	14,904,704	− 61,627,970,099	86,447	77,504	0.072	10,776
	Female	16,977,059	− 75,291,558,959	179,957	162,980	0.127	21,564

*LDCT* low-dose computed tomography, *CXR* chest X-ray, *QALYs* quality-adjusted life-years; *LYs* life expectancy life-years

## Discussion

This study demonstrates that LDCT is cost-savings with the greatest benefits in Japan, but is not cost-effective in the United States. Cost-effectiveness is sensitive to the incidence of lung cancer. LDCT is superior to CXR when the incidence of lung cancer is greater than 0.001 in Japan, one of high-incidence countries. CXR is superior to no screening when the incidence of lung cancer is greater than 0.0002 in the United States, one of low-incidence countries. These advantages are stronger in women than in men. The main reasons for the superior cost-effectiveness of LDCT in Japan are a high detection rate of stage I with a low mortality rate, and a high sensitivity of LDCT compared to CXR. Adenocarcinoma is a predominant histologic subtype of lung cancer in female never smokers [23, 47]. LDCT screening has excellent accuracy in detecting small peripheral adenocarcinomas [48].

To the best of our knowledge, this study is the first cost-effectiveness analysis of LDCT screening for never smokers in the world.

LDCT screening is one of the key screening methods to increase chances of early lung cancer detection with curable treatment and improve health outcomes for never smokers [23, 49]. Sex, age, family history, and the results of the baseline LDCT-scan are also important factors to conduct LDCT screening in never-smokers [49]. The findings of this study suggest that LDCT screening for never smokers, especially women, would be cost-effective in high-incidence countries.

LDCT screening has several disadvantages. Low specificity of LDCT leads to false positive and overdiagnosis. We should be aware of the potential of psychological distress, anxiety, harm caused by false-positive results, and overdiagnosis with unnecessary invasive testing for individuals who choose LDCT screening [50, 51]. Radiation-related cancer by radiation exposure to LDCT screening is also a major concern [52]. Future development of CT diagnostic equipment is expected to reduce radiation exposure during testing.

To introduce population-wise LDCT screening into the national policy, the accuracy of LDCT diagnosis must be ensured regardless of the difference in the physician's capability to diagnose. In the future, artificial intelligence technology will play an important role in ensuring the accuracy of LDCT diagnosis of lung cancer and filling in diagnostic errors. In addition, new biomarkers can help diagnose the malignancy of early pulmonary tumors [53].

The main pathogenesis and carcinogenesis of lung cancer in never smoker are associated with passive smoking history, lung cancer family history, kitchen fume, dust exposure, occupational exposure, radon gas, history of pulmonary infection and tuberculosis, chronic obstructive pulmonary disease, indoor air pollution, ionizing radiation, age, oncogenic viruses, inherited genetic susceptibility, and PM2.5 [54–60].

This study has several limitations. First, we assumed the same stage detection rates of lung cancer in CXR screening and LDCT screening in Japan and the United States. Further prospective cohort studies are needed to determine the differences in lung cancer stage detection rates by LDCT and CXR between Japan and the United States. Second, the age-related increase in lung cancer mortality was not considered in the models. Third, the sensitivity and specificity of LDCT and CXR were not based on meta-analyses [41]. However, we performed one-way sensitivity analyses for the sensitivities and specificities of the screening methods and showed that results were not sensitive to the accuracy of the screening methods. Fourth, the adherence rates of the screening methods were assumed to be 100%. The difference in compliance rates for LDCT and CXR screening was not taken into account. Fifth, we considered only the cost of bronchoscopy with CT-guided lung biopsy as the costs of diagnostic procedures in this model. Sixth, the use of microRNA signatures in whole blood samples complements diagnostic imaging, sputum cytology, and biopsy tests for lung cancer diagnosis [61]. Further cost-effective analysis of microRNAs for lung cancer screening is needed. Seventh, this model does not estimate nonmedical indirect costs, such as reducing the burden on caregivers or reducing productivity. Finally, there are different screening costs and medical systems in each country. Further cost-effectiveness studies by the variance of each country are required.

## Conclusions

LDCT screening for never smokers has the greatest benefits and cost savings in Japan, but is not cost-effective in the United States. LDCT screening reduces lung cancer deaths in never smokers in both countries and saves lives by early detection of adenocarcinomas, especially in never-smoking women. The findings support changing population-based lung cancer screening methods for never smokers from CXR or no screening to LDCT in high-incidence countries. Assessing the risk of lung cancer in never smokers is important for introducing population-based LDCT screening.

## Data Availability

The datasets used and analyzed during the current study are available from the corresponding author on reasonable request.

## Abbreviations

LDCT: Low-dose computed tomography
CXR: Chest X-ray
QALY: Quality-adjusted life-year
LY: Life expectancy life-year
ICER: Incremental cost-effectiveness ratio
NSCLC: Non-small cell lung cancer
EV: Expected value
